# Expanding pneumocephalus due to craniofacial fractures: A case report

**DOI:** 10.1016/j.ijscr.2021.106314

**Published:** 2021-08-16

**Authors:** Mendy Hatibie Oley, Maximillian Christian Oley, Eko Prasetyo, Andreas Suwito, Muhammad Faruk

**Affiliations:** a Plastic Reconstructive and Aesthetic Surgery Division, Department of Surgery, Faculty of Medicine, Sam Ratulangi University, Manado, Indonesia; b Plastic Reconstructive and Aesthetic Surgery Division, Department of Surgery, Kandou Hospital, Manado, Indonesia; c Craniofacial and Cleft Center, Siloam Hospital Manado, Indonesia; d Neurosurgery Division, Department of Surgery, Faculty of Medicine, Sam Ratulangi University, Manado, Indonesia; e Neurosurgery Division, Department of Surgery, Kandou Hospital, Manado, Indonesia; f Neuroscience Center, Siloam Hospital Manado, Indonesia; g Department of Surgery, Faculty of Medicine, Sam Ratulangi University, Manado, Indonesia; h Department of Surgery, Faculty of Medicine, Hasanuddin University, Makassar, Indonesia

**Keywords:** Pneumocephalus, Craniofacial fractures, Pericranial rotational flap, Mount Fuji sign, Case report

## Abstract

**Introduction:**

Pneumocephalus (PNC) is the presence of air in the intracranial cavity. The most frequent cause is craniofacial trauma, though there are many etiologies, including surgical procedures. PNC with compression of the frontal lobes and widening of the interhemispheric space between the tips of the frontal lobes results in the characteristic radiological finding of the “Mount Fuji sign”.

**Presentation of case:**

A 57-year-old man presented to the A&E with loss of consciousness due to a motorcycle collision 9 h prior. He had a GCS of E4M6V5, and a head CT scan revealed minimal PNC forming in both hemispheres. After discharge, severe headaches and rhinorrhea developed. A second CT scan revealed a massive PNC. An operation was then performed via a bicoronal incision to drain the PNC and seal the cranial defect. A burr hole in the calvarium was created, and the cranial defect was closed using a pericranial rotational flap. Post-operation, the patient's headache and rhinorrhea decreased; neither symptom was present at 1-month post-operation. The wounds healed with minimal scarring, and the cosmetic outcome for the craniofacial fracture was acceptable.

**Discussion:**

Although the patient may at first present with a mild head injury, this can progress into something much more serious. PNC is difficult to diagnose clinically. Rarely, patients describe a splashing sound upon moving the head (termed bruit hydro-aerique), which can also be auscultated. A head CT scan is the gold standard in the diagnosis of PNC. Pericranial flaps are widely used for dural repair because they are easily accessible and have a lower rate of infection than artificial grafts on expanding PNC.

**Conclusion:**

Tension PNC may be slow-growing and increase intracranial pressure to high levels before clinical signs are present. The pericranial rotational flap technique is the best way to close a dura mater defect in cranial base fractures with tension PNC.

## Highlights

• Pneumocephalus (PNC) is the presence of air in the intracranial cavity.
• Craniofacial trauma is the most frequent cause of PNC.
• We report a male with expanding PNC who required a pericranial rotational flap.
• Tension PNC may be slow-growing and increase intracranial pressure to high levels before clinical signs are present.

## 1 Introduction

Pneumocephalus (PNC), also termed pneumatocele or intracranial aerocele, is the presence of air in the intracranial cavity. PNC is classified as simple or tension PNC. Other classifications exist based on whether onset is acute (less than 72 h) or delayed (more than 72 h) [1]. The most common cause is craniofacial trauma, but there are many other etiologies, such as surgical procedures, congenital skull defects, infections of gas-forming organisms, neoplastic conditions, and spontaneous appearance [2], [3].

The incidence of PNC depends on the etiology, although it is seen in almost all post-craniotomy cases. The incidence following head injury varies from 1% to as high as 82% depending on the series. This condition was first described by Lecat in 1741, but the term pneumocephalus was first used by Luckett in 1913 and Wolff in 1914. Tension PNC was first described in 1962 by Ectors, Kessler, and Stern. PNC with frontal lobe compression and widening of the interhemispheric space between frontal lobe tips evident in radiological findings is referred to as the “Mount Fuji sign” [4]. Here, following the 2020 Surgical Case Report guidelines [5], we report the case of a male with expanding PNC who required a pericranial rotational flap.

## 2 Case presentation

**Fig. 1:**
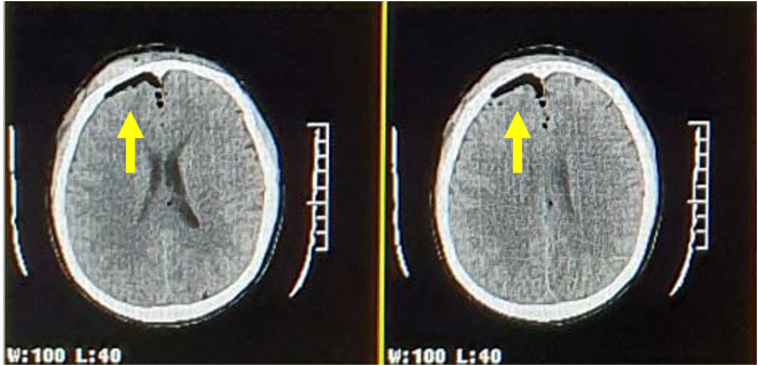
CT scan of brain parenchym window showing minimal right frontal PNC at the first visit to the A&E.

**Fig. 2:**
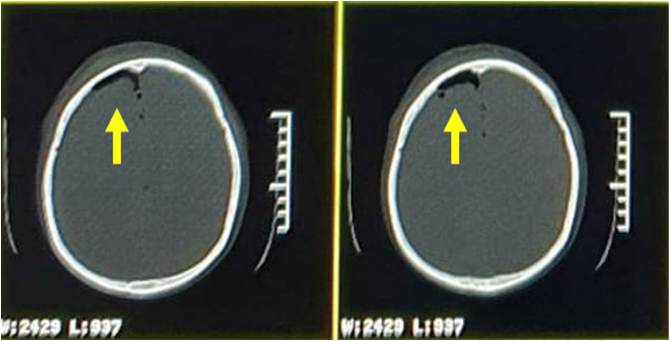
CT scan of bone window showing right frontal PNC.

A 57-year-old man presented to the A&E with loss of consciousness due to a motorcycle collision 9 h prior. Upon initial examination, the patient had a GCS of E4M6V5. The patient had no other symptoms that required inpatient care. The patient also had no history of bloody discharge from the external auditory meatus. The patient's past medical examination shows no other history of past illness and surgery. He reported family history shows the patient's parents died due to heart attacks. Further examination with a head CT scan revealed minimal PNC forming in the right frontal region (Fig. 1, Fig. 2). The patient was first admitted to the A&E then requested for discharge against medical advice after 5 days of inpatient care.

**Fig. 3:**
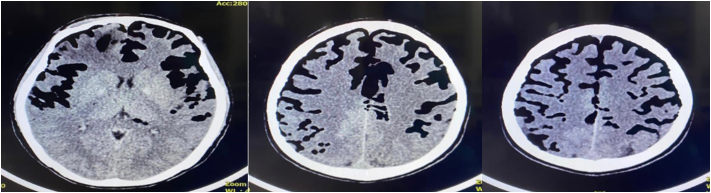
Serial CT scan of brain parenchym window at the second visit showing massive PNC in both frontoparietal lobes.

**Fig. 4:**
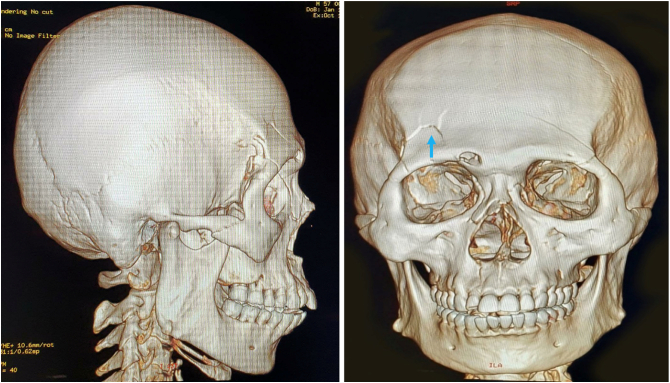
3D Bone Reconstruction showing linear fracture at right frontal lobe.

Ten days later, the patient then came to the outpatient clinic, complaining of severe headache and rhinorrhea developed, prompting the patient to return for re-examination. The patient exhibited right-sided hemipharesa with a GCS of E2V2M4 on the second visit. A second CT scan revealed massive PNC far more severe than evident in the previous CT scan (Fig. 3). Bone reconstruction showed a right frontal linear fracture (Fig. 4).

**Fig. 5:**
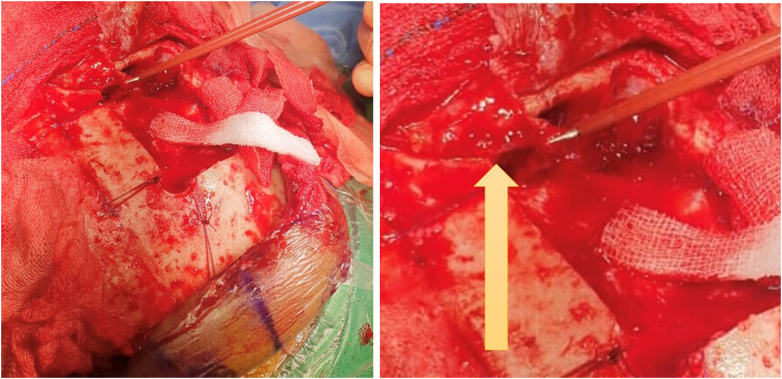
Pericranial rotational flap (arrow).

The final assessment concluded that the patient suffered from a tension PNC. An operation was then performed to drain the PNC and seal the cranial defect. The operation was performed via a bicoronal incision. A burr hole in the calvarium was created at the highest convexity to release the PNC, and the cranial defect was closed using a pericranial rotational flap (Fig. 5). The diameter of the defect was measured, and a flap with the same diameter was drawn adjacent to the defect. The pericranium was sacrificed to obtain clear margins, and the underlying bone was drilled.

He was given an intravenous broad-spectrum antibiotic ceftriaxone (1 g, administered for 5 days) and analgesics drugs (ketorolac, 30 mg intravenous if necessary, not exceeding 5 days). Post-operative was unremarkable, with discharge and rehabilitation taking 6 days for a total of 7 days of inpatient care. Post-operation, the patient's headache and rhinorrhea decreased. The patient was followed up every 3 days for the first week. Sutures were removed 2 weeks post-operative. The patient was then observed every 2 weeks for 3 months. Complaints were minor, consisting of mild headaches. The wounds healed with minimal scarring, and the cosmetic outcome for the craniofacial fracture was acceptable to the patient.

## 3 Discussion

PNC is defined as the presence of air in the cranial vault, typically associated with cranial surgery, craniofacial trauma, nasopharyngeal tumor invasion, or meningitis. Although the patient may at first present with a mild head injury, this can progress into something much more serious [1]. Among pathophysiologic entities, craniofacial trauma is the most common etiology, with as many as 7–9% of patients in this group demonstrating the presence of intracranial air on advanced (CT) imaging [1], [6], [7].

As the result of a head injury or following cranial surgeries, dura may be opened or torn with or without injury to the arachnoid mater. In such cases, air may enter the cranial cavity. Air is a source of infection that can lead to the development of meningitis [1], [4].

PNC is difficult to diagnose clinically. Rarely, patients describe a splashing sound upon moving the head (termed bruit hydro-aerique), which can be auscultated as well. X-rays have been used in the past to identify PNC, but these will miss small quantities of air. A head CT scan is the gold standard in the diagnosis of PNC. It can detect even 0.55 ml of intracranial air, whereas a skull radiograph requires at least 2 ml. Air has a Hounsfield coefficient of −1000. Ishiwata et al. identified two signs as characteristic of TP. “Mount Fuji sign” (named after Mount Fuji, the highest volcanic mountain in Japan) is formed by the accumulation of air in the frontal region, with separation of the tips of the two frontal lobes. This radiological finding in a patient in the supine position is diagnostic of tension PNC. “Air bubble sign” denotes the presence of multiple air bubbles scattered in several cisterns. “Peaking sign” denotes bilateral compression of the frontal lobes without separation of the tips, indicating a less severe condition compared with Mount Fuji sign [4], [8].

Treatment of simple PNC is typically conservative. It involves bed rest, placing the patient in a 30-degree Fowler position, avoiding Valsalva maneuvers (e.g., nose-blowing, coughing, and sneezing), administration of analgesics and antipyretics, and osmotic diuretics. If indicated, high-flow oxygen therapy should be administered (5 L per minute for five days, at least) via a face tent or 100% non-rebreather mask with absolute avoidance of positive pressure [7], [9].

Pericranial flaps have been used for the repair of dural defects for more than 30 years. A pericranial flap is an example of a collagenous autograft. This autograft may have preferable biologic responses compared with synthetic grafts, including reduced inflammatory responses. In addition, it may be possible to remodel with host cells and native vasculature. The pericranium has a dual blood supply, directly from peripheral vessels arising from the internal and external carotid arteries, as well as from perforators to the galea. Although galeal perforator supply is necessarily lost during graft harvest, maintenance of a pedical allows preservation of vascular supply to the tissue. Pericranial flaps are widely used for dural repair because they are easily accessible and have a lower rate of infection than artificial grafts. Vascularized flaps increase the rate of successful dural closure and minimize the risk of cerebrospinal fluid leak and infection. Smaller defects are often successfully addressed with local two-layer pericranial or three-layer myofascial galeo-pericranial flaps. However, larger cranial base defects require supporting materials in addition to local flaps for a more rigid construct [9], [10].

## 4 Conclusion

Tension PNC may be slow growing and increase intracranial pressure to high levels before clinical signs are present. A pericranial rotational flap is the best way to close a dura mater defect in cranial base fractures with tension PNC. However, as shown here, clinical findings may evolve beyond those present at first examination. Here, we see port d'entree from the facial fracture slowly causing massive, possibly life-threatening PNC.

## Provenance and peer review

Not commissioned, externally peer-reviewed.

## Funding

This study did not receive any specific grant from funding agencies in the public, commercial, or not-for-profit sectors.

## Ethical approval

The study is exempt from ethical approval in our institution.

## Consent

Written informed consent was obtained from the patient for publication of this case report and accompanying images. A copy of the written consent is available for review by the Editor-in-Chief of this journal on request.

## Registration of research studies

Not applicable – single case report.

## Guarantor

Mendy Hatibie Oley.

## CRediT authorship contribution statement

Mendy Hatibie Oley, Andreas Suwito, and Maximillian Christian Oley: study concept and surgical therapy for this patient. Mendy Hatibie Oley and Andreas Suwito: data collection and writing-original draft preparation. Maximillian Christian Oley and Eko Prasetyo: senior author and the manuscript reviewer. Muhammad Faruk: editing and writing. All authors read and approved the final manuscript.

## Declaration of competing interest

Nothing to declare.
